# Miscellaneous

**Published:** 1874-09

**Authors:** 


### MISCELLANEOUS.

### FREEZING CELLARS.

A FARMER prevents frost in his cellar by pasting the walls and the ceiling over with four or five thicknesses of old newspapers, a curtain of the same material being also pasted over the small, low windows at the top of the cellar. The papers were pasted to the bare joists overhead, leaving an air space between them and the floor. He reports that the papers carried roots through last winter, though the cellar was left unbanked, and he is confident they have made the cellar frost-proof. Whatever paper is employed, it will be necessary to sweep down the walls thoroughly, and use a very strong size to hold the paper to the stones. It is not necessary to thrust the paper down into all the depressions of the wall; every air space beneath it is an additional defence against the cold.

No doubt cellars may be kept warm in the manner proposed, but it is at the risk of burning the house down. Cellars are often visited after night with lamps or lighted candles. No inflammable material should ever be allowed in a cellar, for owners as well as servants are sometimes careless.

---

### AN INCIDENT IN THE CARS.

ON the whole, pleasant traits and characters are not common in the cars. This opinion I expressed to my friend Summers the other day. In reply to my remarks he related a little adventure, which, as it is *apropos*, and moreover involves a little love and sentiment, I give it without apology, and in his own words. It appears that in the most unlikely places love and sentiment may be discovered.

"I was escorting home the lovely Charlotte D., to whom I was at the time quite devoted. Charlotte could scarcely find room to spread her voluminous flounces. I stood up near her, there being no vacant seat.

"After a few minutes came in a poor woman, who deposited a basket of clothes on the front platform, and held in her arms a small child, while a little girl hung to her dress. She looked tired and weary, but there was no vacant seat; to be sure Charlotte might have contracted her flounces, but she did not. Beside her, however, sat a very lovely and elegant young woman, who seemed trying, by moving down closer to others, to make space enough for the stranger between herself and Miss D. At last she succeeded, and, with the sweetest blush I ever saw, she invited the poor female to be seated. Charlotte D. drew her drapery around her, and blushed too, but it was not a pretty blush at all, and she looked annoyed at the proximity of the new comer, who was, however, clean and decently though thinly clad.

"The unknown lady drew the little girl upon her lap, and wrapped her velvet mantle around the small, half-clad form, and put her muff over the half frozen little hands.

"So great was the crowd that I alone seemed to observe. The child shivered; the keen wind from the door blew upon her unprotected neck. I saw the young lady quietly draw from under her mantle a little woollen shawl, which she softly put on the shoulders of the little one; the mother looked on with confused wonder. After a short time she arose to leave the cars, and would have removed the shawl, but the unknown gently whispered, 'No, keep it for her.' The woman did not answer, the conductor hurried her out, but her eyes swam with tears. I noticed her as she descended to a basement, and I hastily remarked the house.

"Soon after my unknown rose to depart. I was in despair, for I wanted to follow and discover her residence, but could not leave Miss D.

"How glad, then, I was to see her bowing, as she passed out, to a mutual acquaintance, who stood in the doorway. From him, ere many minutes, I learned her name and address.

"To shorten the story as much as possible, that lady is now my wife. In the small incident which introduced her to me she showed her real character. A few days after our marriage I showed her the blessed crimson shawl, which I redeemed from its owner, and shall keep as a memento. There are sometimes pleasant things to be found in unexpected places; certainly, I may be said to have picked out my wife in the cars."

*Review, Baltimore.*

---

### THERE.

Do any hearts ache there, beyond the peaceful river?
  Do fond souls wait, with longing in their eyes,
For those who come not—will not come forever—
  For some wild hope whose dawn will never rise?

Do any love there still, beyond the silent river,
  The ones they loved in vain this side its flow?
Does the old pain make their heart-strings ache and quiver?
  I shall go some day, go home and know.

The hill tops are bright there, beyond the shining river,
  And the long, glad day, it never turns to night;
They must be blessed, indeed, to bear the light forever;
  Grief longs for darkness to hide its tears from sight.

Are tears turned to smiling beyond the blessed river,
  And mortal pain and passion drowned in its flow?
Then all we who sit on its hither bank and shiver,
  Let us rejoice—we shall go home and know!

### WHAT KILLED THE "INGHAM COUNTY HERALD."

Fifteen or sixteen years ago a man named Harvey started a weekly paper at Williamston, Ingham County, called the *Herald*. The town was small, the times dull, and Harvey was probably the laziest editor who ever had anything to do with a Michigan paper. The citizens encouraged him all they could, and finally the gentleman who had furnished most of the capital dropped into the office one Friday to see Harvey and spur him up a little. It was publication day, but the outside, only half made up, was on the stone, and not a line had been set for the inside. Harvey was in the back yard of the office, digging fish worms, and was called in.

"See here, Harvey, you are not doing as you should," commenced the gentleman; "here it is Friday, and you haven't even worked the first side of the paper."

"I—see—I haven't!" slowly replied the editor, looking in the drawer for a fish line.

"Well, when are you going to get the paper out?"

"Some—time—next—week."

"But this is no way to do business, Mr. Harvey. When you came here didn't you agree to issue the paper every Friday?"

"I—presume—I—did," drawled Harvey, "but—I—didn't—know—that—fishing—was—so—good."

He took his rod and went to the banks of the Cedar, and when he returned the office was packed up in boxes, marked "old type," and a note on the door read: "The *Herald* is dead—too many suckers killed it."—*Detroit Free Press.*

---

### A MATRIMONIAL PROJECT.

Mayor Johnson has received a touching letter from a New Orleans gentleman. The writer states that he has been in correspondence with Miss ——, residing at No. — L—— street; that the correspondence promises soon to reach the awful climax of marriage; that the happy lover of the sunny South contemplates coming to Cincinnati soon to see his lady and wed her, and he wants to know of the Mayor what sort of a woman she is, anyhow. He doesn't want to go to the expense of buying new clothes and paying his way here if everything isn't right on the goose. Good references are given, including the Mayor and other prominent citizens of New Orleans.—*Cincinnati Commercial.*

---

### ORIGIN OF THE FORGET-ME-NOT

This popular tradition, which tells how the name came to be applied to the plant which now bears it throughout Europe, is not generally known. It is said that a knight and a lady were walking by the side of the Danube, interchanging vows of devotion and affection, when the latter saw on the other side of the stream the bright blue flowers of the myosotis, and expressed a desire for them. The knight, eager to gratify her, plunged into the river, and, reaching the opposite bank, gathered a bunch of flowers. On his return, however, the current proved too strong for him, and, after many efforts to reach the land, he was borne away. With a last effort he flung the fatal blossoms upon the bank, exclaiming as he did so, "Forget-me-not!"

### THE IMPUDENT MAN

The genuinely impudent man—the man with a supreme incapacity for feeling or showing shame—moves through the world graceful and serene. No one calls his impudence impudence; it is generally *savoir faire*, tact, good style—the spade is anything, in short, except a spade. He is satisfied and at ease with himself, and, therefore, makes others satisfied with him and with themselves. The so-styled man is the shy man, who being conscious to himself of real or imaginary inferiority, thinks himself obliged to seem what his unhappy lack of true impudence and true modesty will not allow him to be. Being in his heart afraid of his own voice, he rushes into talk with the random rashness of a coward.. Believing himself to be necessarily underrated, he seizes his own trumpet and blows thereon the wearisome note of I. He thinks it a social duty to be entertaining, always and everywhere, and so, like the German Baron, practices "how to be lifely" by morally jumping over the tables and chairs. Afraid or ashamed of being left in the rear, he makes a point of charging in the very van. And thus, while the impudent float quietly and securely down the stream, he throws himself into social whirlpools for fear of being drowned.

---

### A GLIMPSE OF SPANISH LIFE.

All the Spaniards rise, as a rule, at five or six in the summer to enjoy the only enjoyable time of the summer day; at one o'clock they have dinner—the comida—and after that follows the two hours siesta in the darkened room. Evening then draws on, the delicious light breeze rises and blows freshly from the hills, and the ladies go out in groups to the alemedo for the passao, or walk. Such is the Spanish lady's day. She has, however, her creadas to look after; and, above all, her dresses to make or superintend, and her graceful mantilla to arrange. It is quite a striking sight to pass down the streets, from six to eight at night, and see the graceful carriage of the head, and the stately upright walk of the Spanish ladies, with their long, white dresses trailing behind them in a cloud of dust. How they manage to walk over the rough, unpaved, uneven streets without a trip is a mystery. At about ten all retire to rest, to rise up refreshed for another eventful day. As regards the master of the house, he really seems to have but one interest in life, and that is politics. He may ride out to view his olive farm or hill mine; and you will certainly meet him in his shop, his casino, or his friend's casa, smoking the inevitable cigarillo, and chatting, or making a bargain. But there is absolutely no reading of any sort, not even a book of the calibre of a three volume novel. Politics, politics are everything to him, and of politics he seems never to tire.—*McMillan's Magazine.*

---

According to a French veterinary surgeon, a simple method of preventing flies from annoying horses consists in painting the inside of the ears, or any other part especially troubled, with a few drops of empyreumatic oil of juniper. It is said that the odor of this substance is unendurable to flies, and that they will keep at a distance from the parts so anointed. If this treatment should accomplish the alleged results, it may, perhaps, be applicable in repelling mosquitoes from the faces and hands of tourists and sportsmen when passing through woods or meadows.

### A NEW CHINESE COMPOSITION.

Dr. Scherzer, an Austrian official at Pekin, has sent to his Government some specimens of a Chinese composition called "Schioicao," which has the property of making wood and other substances perfectly water-tight. He says that he has seen in Pekin wooden chests which had been to St. Petersburg and had come back uninjured, and that the Chinese use the composition also for covering straw baskets, which are afterward employed for carrying oil long distances. Card board, when covered with the composition, becomes as hard as wood, and most wooden buildings in Pekin have a coating of it. It consists of three parts of blood, deprived of its fibrin, four of lime, and a little alum.

### AN EFFEMINATE MAN,

Says a recent writer, is a weak poultice. He is a cross between table beer and ginger pop, with the cork out; a fresh water mermaid found in a cow pasture, with her hands filled with dandelions. He is a teacup full of syllabub; a kitten in trousers; a sick monkey with a blonde moustache. He is a vine without any tendrils; a fly drowned in oil; a paper kite in a calm. He lives like a butterfly—nobody can tell why. He is as harmless as a pennyworth of sugar candy, and as useless as a shirt button without a hole. He is as lazy as a slug, and has no more hope than last year's summer fly. He goes through life on tiptoe, and dies like cologne water spilt over the ground.

### HOW THE EYE IS SWEPT AND WASHED.

For us to be able to see objects clearly and distinctly it is necessary that the eye should be kept clean. For this purpose it is furnished with a little gland, from which flows a watery fluid (tears), which is spread over the eye by the lid, and it is afterwards swept off by it, and runs through a hole in the bone to the under surface of the nose, while the warm air, passing over it while breathing, evaporates it. It is remarkable that no such gland can be found in the eyes of fish, as the element in which they live answers the same purpose.

If the eye had not been furnished with a liquid to wash it, and a lid to sweep it off, things would appear as they do when you look through a dusty glass. All along the edges of the eyelids there is a great number of little tubes or glands, from which flows an oily substance, which spreads over the surface of the skin, and thus prevents the edges from being sore or irritated, and it also helps to keep tears within the lid. There are also six little muscles attached to the eye which enable us to move it in every direction; and when we consider the different motions they are capable of giving to the eye, we cannot but admire the goodness of Him who formed them, and thus saved us the trouble of turning our heads every time we wish to view an object.

### THE HEROINE OF THE BORDER.

It was a lonely night in August, seventy years ago. The sky had not a cloud; the air was soft and balmy; and the moon, sailing on high, flooded the field and river with her calm, pure light. Never, since the last evening in Eden, had mother Earth worn an aspect so like Paradise.

At the gate of a mansion, in the then newly settled town of Wheeling, stood two lovers, about to part. The solemn spell of the hour, combined with the farewell they were about to take, had cast a sweet sadness over both.

"It is a long ride to Shepherd's Fort, and I almost fear for you," said a low and now timid voice, as the fair speaker looked into the face of her companion. —"Can you not put off this departure, dear Francis?"

The bold Virginian gazed tenderly down at his beautiful companion, and then, as if instinctively, drew her to him, imprinting a kiss on her forehead, as he answered:

"You were not always thus timid, Lizzy. I remember before you went to Philadelphia, that you were the boldest girl of all the border; but to-night, you are as fearful as a young doe. You have come back accomplished as a queen, but as nervous too. A fine lady," he continued, laughingly, "it is not for a bride on the frontier."

"I am not naturally timid as you know," earnestly replied his companion, blushing at his raillery; "but these terrible Indians appal me. You tell me that they have held a great council at Chillicothe, and resolved to take up the hatchet again. What if a band of them should waylay you to-night."

"Never fear it, dearest; they are far enough as yet. My information is in advance of them, and we shall probably not see them for weeks, even if at all. I thought it my duty to ride over and tell your father, however; though," he added archly, "perhaps there were other reasons which made me only too ready to find an excuse for visiting Col. Zane."

"I suppose my fears are foolish," answered his companion; "but with woman, instinct, they say, sometimes takes the place of reason; and I feel as if some great danger surrounded us."

"It is the moonlight, Lizzy, nothing else," gaily replied the lover. "I always feel a touch of sadness myself on an evening like this. Cheer up, dearest; these are idle fancies. I must, however, make the most of this moonlight, or it will be dark before I reach the fort. God bless you," he added, as he kissed her again, adding bravely, "if peril should assail you, not ten thousand times ten thousand devils shall prevent Francis Duke flying to your aid."

In another moment he had leaped upon his horse, which stood hard by, and, with a parting wave of the hand, dashed off at a swinging trot. His betrothed watched him till he disappeared in the gloom of the forest; then, with a sigh, she re-entered her father's house.

The family was about retiring, and reverently kissing her parents, the beautiful girl ascended to her chamber. But she could not sleep. An indefinable sense of danger pressed upon her spirits, and throwing aside the curtain of her window, she sat down and gazed without. As she looked, a vision rose before her of her lover, hurrying along the forest depths, while a savage ambuscade waited to receive him; and so vivid was the picture, that she rose with a start of terror, and could hardly repress a cry. But the next instant the illusion faded, and the serene moonlight calmed her soul, and she resumed her seat, ashamed of her idle terrors.

Thus, hour after hour passed, her fancy continually conjuring up new perils for her lover, and her sober reason as constantly overcoming these visionary fears. Her sleeplessness continued. At last the moon began to sink behind the western forest. Slowly she watched the lengthening shadows as it disappeared, until the landscape was almost entirely shrouded in gloom; and then, with a last prayer for her lover's safety, she was about to retire from the window when her attention was suddenly attracted toward what seemed a dark moving mass on the edge of the neighboring woods.

What could it be? The cattle of the settlement were all carefully housed, she knew, and it was an hour at which no human being would be likely to be abroad. Her indefinable fears again returned.—Her heart fluttered with nameless dread. She concealed herself instinctively behind the window curtain, and watched the dark, moving object for several moments, until finally it emerged from the shadow of the forest, when its uncertain outlines resolved themselves into those of two Indians in their war paint.

The curtain fell from her hands and she sank into her chair, completely unnerved. All her late tremors were now explained. The hostile savages, instead of being far distant, were close at hand. The very warriors she had just seen might have intercepted her lover; and his scalp might be even now at the girdle of one of them. At this horrible idea she clasped her hands shudderingly over her eyes, and, for an instant, forgot her misery in oblivion.

But this lasted only for a moment—Lizzy Zane was no weak, timid girl, whom danger rendered a burden to her friends; on the contrary, hers was a soul cast in the heroic mould of Joan of Arc, and other of the brave and dauntless of her sex. Hastily springing to her feet, she cried; "This tremor is childish. What if Francis is dead, since peril surrounds my parents, and not only them, but the whole settlement. The Indians have waited till the moon was down to commence their attack, hoping to surprise us; and the two I have seen doubtless outlaying scouts, who fortunately for us have shown themselves too soon. I will rouse father at once. It will be time to weep for the dead, when my duty to the living is fulfilled."

With these words she hurried from the chamber, though not till she had taken another guarded look from the casement. This time not a living soul was in sight. She rightly judged that the scouts had gone to bring up their companions, and lost no time consequently in arousing the household.

At that period in the history of the West, every frontier man slept, as it were, on his arms. But the present summons was so unexpected, and the assault of the savages might be looked for so soon, that preparations for defence could not be made with the usual care. Col. Zane, on being aroused, dispatched servants to summon his neighbors to the contiguous fort, which stood about forty rods from his own dwelling. About twenty men answered the call, bringing with them their families, but necessarily abandoning their stocks and everything else to the chances of war. He determined himself to hold his mansion, it being favorably situated as an outpost, and containing large stores of ammunition; accordingly retaining three men to assist him, he ordered the remainder of his people to repair to the fort. In vain did his daughter entreat to remain with him; he bade her to remember that while duty bade him stay, it would be criminal temerity for her to remain; and, deluged with tears, she obeyed his commands.

Scarcely had the gates of the fort been closed, less than half an hour had elapsed since the first alarm, when the army of savages appeared emerging from the forest. It was not long before they saw that their approach had been discovered, and that the fort was in readiness to receive them; so, with a yell of rage and hate, they rushed to the assault.

"Keep cool, my men," said the commander of the fort, an uncle of our heroine, "and fire only when you can clearly make out an Indian. It is easy to confound these dusky devils with the shadows: and this they know; so take care and waste no ammunition. The women will load for you, when the hellhounds come to close quarters, and quick firing is necessary; fortunately we have plenty of muskets, so that fresh ones can be handed to you as fast as you require them. I will set you an example, and my niece, God bless her, will load for me and show how an American woman can assist in the hour of peril."

His words were cut short by the necessity for instant exertion. The Indians had, by this time, crossed the field in front of the fort, and were approaching, partly under cover of the trees, towards the entrance, hoping to force their way in. Instantly from the house of Col. Zane, as well as from the fort, a heavy discharge of musketry was opened on the savage assailants. Every man, in each edifice, knew that he was fighting, not only for his own life, but for those dearer than life. Their wives, daughters, and other female relatives, conscious of the same truth, heroically assisted them, loading the muskets as they were discharged, and praying silently for aid from heaven.

"Ha! they recoil," shouted the commandant, "the red skins can't stand the firing of desperate men. The colonel is giving it to them gallantly from his house. Hark! that is his hurrah. Let us give him back a cheer. That was a true Virginia shout. God be praised; the devils run. Huzza for victory."

With exclamations like these, the old soldier sustained the spirits of his men during this short but terrific conflict, until, as his last words betokened, the assailants were in full flight; and then another shout went up from the fort, which was answered back as sturdily from the outpost, where, with his three assistants, Col. Zane kept his dwelling against the savages. It was worth peril unto death to her and to participate in that huzza.

But if the defenders of Wheeling flattered themselves that they had achieved a permanent triumph, they were soon destined to discover their mistake. The Indians, though repulsed, were not defeated, and, after a slight interval, were seen again rushing to the assault. Again, at the commandant's injunction, the men withheld their fire till the savages were close to the wall; again the muskets blazed in an almost continuous stream; again the women moulded bullets and loaded the still smoking guns; and again the iron like voice of the commandant rose over the sharp crack of the rifles, encouraging his subordinates, while the loud huzza of Col. Zane answered back from the outpost, as if he laughed at, and even rejoiced in the peril. Meantime, on the side of the assailants, the utmost fury prevailed. The loss of many of their number had excited them to apparently demoniac madness, and their yells, always hideous, sounded like those of fiends let loose on earth. Their dusky painted forms, dimly seen by the red glare of the muskets, flitting hither and thither as they dodged from shelter, in their approaches, adding to this horrible illusion.

"Blaze away, my lads," shouted the commandant, "for they begin to waver, and another volley or two will send them to the right about. Ha! that red devil had his death shot from my brother's rifle; I know the crack of the old piece.—That's a brave girl, Lizzy; you load to perfection. Now for that cursed Wyandot, that is climbing over the paling; I have my bead on him; there the rascal tumbles. And see, appalled by his fall, the rest are making off. Huzza, huzza, huzza!"

It was as he said. Headed by a young chief, the Indians had suddenly broke their coverts in front, and dashed like a pack of hounds, fierce for blood, at the palisades. Their leader had reached this destination first, and leaping, as would a deer, had gained the top at a single bound, when the rifle of the commandant covering him, in an instant after, he fell dead within the fence. His death was the signal for his followers to retreat, terror struck, in all directions, an event which was again hailed by a shout from the fort, and an answering huzza from the outpost.

"There, we are safe for this night at least," said the commandant, bringing the breach of his rifle to the floor. "I never knew the red-skins to make two assaults before, in darkness; and the devil is in them if they risk a third. But, at daybreak, look out. We shall have them on us again, at that hour, in all their force, and howling like ten thousand fiends incarnate. Such of you as would like to sleep can do so; it will be easy to awake you, if necessary."

But all were too anxious to sleep.—From the loop-holes of the fort, eager eyes continually watched the distant wood, in which the savages had sheltered themselves, in order to detect the first movements of the foe. The prediction of the commandant, however, proved correct.—No further assault was ventured that night. But an attempt was made to destroy, by stratagem, the house of Col. Zane, the fire of whose defenders had been, from its proximity, so annoying to the Indians. For this purpose a savage crept up to it with a lighted brand; but watchful eyes were on the foe in the outpost as well as in the fort; and, just as the Indian was about to apply the torch, a shot sent him howling away.

During this cessation of the strife, the thoughts of our heroine again recurred to her lover. But her uncle, who perhaps suspected her fears, cheered her by assurance that he had escaped—

"Nay, look not so dull, Lizzy," he said, "now that the battle is over. Frank is safe, before this, at Shepherd's Fort. His route lays in the opposite direction to that by which these devil's clearly came. The peril will be for him to-morrow, when he attempts to join us, for I know nothing of him, if he don't gallop to our aid, even if he does it alone."

The dawn was now approaching. With the first gleam of light, the savages were seen to be in motion; but they did not risk a close assault, as they had done in the night; they sheltered themselves at a distance, and opened a desultory fire on the fort and outposts, seeking to pick off the garrison one by one.

The men, however, kept themselves carefully concealed, so that none were hurt; but, on several occasions, a savage who had momentarily exposed himself, met his death.

An hour passed in this desultory warfare when the commandant, looking forth, said:

"What can the red-skins be at now? Here, Lizzy, your good eyes are sharper than my old ones. Have they a wagon there?"

"It looks like a cannon, only the Indians never have such things. They are wrapping something around it. Ah! now I see; it is a hollow log, and they have taken some chains from the shop, with which they are binding it. And there, too, are cannon balls."

"Now I understand it," said the commandant, laughing. "Jim Stokes, who found his way into the fort just before daybreak, had a load of cannon balls in his flat boat, when the savages surprised him last night. The fools, I suppose, are about to fire them at us from their hollow logs! Batter us down with wooden cannon! Ha, ha, ha! I fancy we shall first see some of the red-skins blown to kingdom come."

The Indians had by this time finished their preparations, and, almost immediately after, the crowd opening from around their impromptu gun, a match was applied to it. As the commandant had foretold, the crazy weapon exploded, scattering death and terror among the throng. The first effect was to paralyze the savages: the second was to rouse them to phrenzy. Breaking into yells of rage, they burst from the spot, taking the direction of the fort, evidently determined to avenge the death of the slain even if they perished themselves.

"Now, my boys, comes the final struggle," said the commandant. "If we beat them back this time, the victory will be complete; but if a single trigger fails, our scalps will be drying, before a month, in the Wyandot lodges. Think of your wives and children !"

He was already taking his station, when his niece silently touched his arm. Her face was full of perplexity and even terror; and for the first time since the siege had begun.

"What is it my child ?" quickly asked the commandant, his countenance assuming something of the look of hers, for he was aware that no common incident could have worked such a change in his niece. And he drew her to one side, saying "speak in a whisper."

"The ammunition is out, unless there is more hid away than you gave into my charge."

A blank settled on the commandant's countenance.

"Good God, he exclaimed, it is all over with us, for we have no more in the fort. I brought what I thought enough, from your father's house, but these repeated assaults have consumed it."

"Cannot some one," said the niece, "go to our house and bring back to us a supply ?"

"But who ? It would be almost certain death."

There was a moment's pause. Then, with no change in her countenance, except that it grew a shade paler, she answered : "I will go !"

"You !" said the veteran starting back.

"And why not ? I am young, active, and fleet of foot. I can go quicker than any one else. Besides savages, seeing it only a woman, may not fire till they detect my purpose ; and in that case only the return will be perilous. If I do not go we are all sure to die. Even if mortally wounded, I think I can succeed in gaining the fort again, and if this can be done, and your lives saved, I am content to fall."

"But Frank——"

For a moment her lip quivered. She grasped her uncle's arm convulsively, gave him an appealing look, and said, in a husky voice—

"Don't—don't. Let me go before it is too late."

The tears came into the old man's eyes. He pressed his niece to his bosom, and then said :

"Go, then, and God be with you, heroic girl ! And yet," he added, glancing around, "If it were not for these poor women and children, neither I nor any man here would suffer it."

When the intention of our heroine was made known, several of the men insisted on taking her place ; but her reply was :

"Not one of you can be spared—a woman will be less missed."

Sadly the little garrison beheld her depart on her terrible venture. Her uncle himself, notwithstanding the danger, attended her to the gate of the fort, which he himself threw open ; and with the fleetness of a young fawn, away she bounded.

The defenders of the outpost saw her departure, and though ignorant of its cause stood ready to receive her. Col. Zane in person hurried to the door. The savages, too, beheld her exit. At first a dozen guns were levelled at her, but when it was seen that the fugitive, as they thought a contemptuous cry of "a squaw, a squaw" passed from one guttural throat to another, and she was suffered to gain the dwelling unharmed.

"What is the matter, my child ?" cried her father, as she dashed into the doorway.—"Why do you madly risk your life ?"

"We are out of powder," breathlessly answered our heroine. "All is lost unless I can bring a supply. Give me some quick, or the Indians will have made their last rush." And as she spoke she sank almost exhausted, on a chair by the door.

For an instant her father regarded her in silence. Wonder, tenderness, and admiration succeeded each other in his heart, and were depicted in his face. The whole peril of her proceeding rose before him. Her return he well knew wou'd be through a gauntlet of balls which the savages on detecting her purpose would pour upon her. Escape with life would be almost impossible. She was his only daughter too; it would break his heart to lose her.

But though human weakness whispered all this, tempting him to keep her from returning, his sense of duty triumphed; and with but a single moment's delay, he snatched a table cloth, which he himself fastened around her waist, while, in answer to his eager calls, one of his companions brought a keg of powder and poured it in. Then snatching a hasty kiss, he threw open the door, and with a voice heroically calm, and a look like that of Abraham when about to sacrifice his only son, he cried:

"Run for your life, and God help us both!"

Away she bounded again, this time fleeter than before, if that were possible. Her appearance was hailed by the savages with a storm of yells, for, with the craft of their race, they instantly divined her purpose. A score of rifles simultaneously were brought to shoulder. Some of the Indians, fearful she might escape, fired at once.

The balls whistled close by, but she was unharmed. Others took aim, and the shots from these now began to sing past; but still, as if protected by a miracle, she bounded on apparently unhurt. Her father stood watching her, too absorbed to speak, but his heart beating like a forge hammer. Nor was her uncle less dumb with emotion. Others, however, were not so breathlessly silent.

"See, cried one of the garrison, that ball went through her apron; the powder pours through the hole. She darts forward like an arrow. The shots rattle around her like hail, but she is still unhurt. Only a few rods remain. Heavens, she is struck. No, she only tripped a little—she is up again; the stumble has, perhaps, saved her life, for the rifle balls went over her like pigeons on the wing. Huzza, she gains the gate, she is safe. Thank God, thank God." And the weather beaten borderer burst into tears of mingled joy and excitement, in which the women loudly shared, as they rushed to welcome our heroine back.

Her uncle, who had hurried to the gate to meet her, now brought her forward.

"Yes," he cried, "hang around her, embrace her, thank her, for she has saved all our lives. There, that is enough; to your posts, all of you; for the yell I hear announces that the savages are renewing the assault. But ha! what is that?" he cried, as taking up his place at the loop hole, he looked out. "A horseman dashing toward the gate, and Frank Duke as I live. He cries to open to him. A shower of balls are falling around him. Run, Lizzy, and let him in.

He turned to address his niece, but she had already darted to the gate. Well was it that she had been so quick, for just as she flung open the portal a shot struck her lover and he fell headlong from his steed. The savages were close behind, and, mad with so many failures, seemed bent on securing his scalp at every hazard; a dozen, indeed, were already within as many yards of the gate. The hesitation of a moment would have lost all. But, in that crisis, our heroine's presence of mind did not desert her; she rushed forward outside the gate, seized Frank by the arm, and with superhuman energy dragged him staggering within the gate; then letting him fall, hastily closed and barred the portals. As she placed the huge piece of wood across the doors, she felt them quivering with the tomahawks hurled at her and Frank, in the last effort of baffled rage, but the thick planks were between her and the deadly weapons, and she knew well the peril was past. She fell on her knees at this conviction, beside the body of her lover, and audibly thanked God.

The fire from the fort and outpost, meantime, had been most deadly; the last rush of the savages had exposed themselves to great losses; and, almost simultaneously with the rescue of Frank, the assailants fell back. The siege, in fact, was now over, as the commandant had predicted it would be. But it was not till the third day that the savages finally broke up from before the fort and returned across the Ohio.

Before that time Frank was out of danger. At first his wound had been considered mortal; but assidious care and strong constitution saved his life. His return he explained as soon as speech was restored to him. He had fallen on the trail of the enemy just before daybreak, and finding that it ran in the direction of Wheeling, had put spurs to his horse and galloped to the aid of the scanty garrison.

The sequel to our tale may be imagined. The heroine and her lover were, in due time, united. Never did the Old Dominion behold a nobler couple, though many have been her beauteous brides and numerous her gallant bridegrooms. And history, we may add, may be searched in vain for a deed of female heroism to surpass that which the siege of Wheeling witnessed.

### THE JOKE OF A STAGE DRIVER.

Hank Monk, the stage driver who gave Horace Greeley his memorable mountain ride, in common with all his tribe, hates the sight of those ponderous specimens of architecture in the trunk line known as the "Saratoga band-box." He likes a "Saratoga" about as well as a cat likes hot soup. He now drives on the stage line between Carson City and Lake Tahoe. He was driving on the same line last summer. A Virginia lady, who was stopping at the Glenbrook House, had a "Saratoga" at Carson, which she wished brought up to the lake. It was about as long and wide as a first-class spring mattress, and seven or eight feet high. The lady had managed to get it as far as Carson by rail, but the trouble was to get it up into the mountains. Hank had promised two or three times to bring it up "next trip," but always arrived without it. At last Hank drove up one evening, and, as usual, the lady came out upon the verandah to ask if he had brought her trunk. Like the great and good man, George, Hank cannot tell a lie, and so he said :

"No, ma'am ; I haven't brought it ; but I think some of it will be up by the next stage."

"Some of it !" cried the lady.

"Yes ; maybe half of it, or such a matter."

"Half of it !" groaned the horrified owner of the Saratoga.

"Yes, half to-morrow, and the rest next day or the day after."

"Why, how in the name of common sense can they bring half of it ?"

"Well, when I left they were sawing it in two, and—"

"Sawing it in two ! Sawing my trunk in two !"

"That was what I said," coolly answered Hank. "Two men, with a big cross-cut saw, were working down through it — about in the middle, I think."

"Sawing my trunk in two ? and all my best clothes in it ! Sawing it in the middle ! God help the man that saws my trunk !" And, in a towering passion, she rushed indoors, threatening the hotel, the stage line, the railroad company, the town of Carson, and the State of Nevada with suits for damages. It was in vain that she was assured that there was no truth in the story of the sawing—that Monk was a great joker ; she could not be made to believe but that her trunk had been sawed in two until it arrived intact, and she had examined its contents most thoroughly.

Monk's "Saratoga" joke is still remembered and told at the Glenbrook House, but the ladies see no fun in the yarn.

Special Notice.—We will send any $4.00 Paper or Magazine for one year, and "Hall's Journal of Health and Miscellany" six months, for $4.00.

### THE WAYSIDE VISION.

BY THOMAS DURFEE.

On the sidewalk cool and shady
Five paces in her rear I strolled,
She was a lithe and beauteous lady,
A sylph in motion and in mould ;
And moved past all the fret and fever
Of earthly toil for earthly gain,
Like one whose sunny heart had never
Felt a breath of earthly pain.

Around her form the shadow lingered,
Like some fond wooer loth to go,
And with caresses, rosy-fingered,
The sunlight flitted to and fro ;
And where the bolder zephyr hovered,
Won favors from her neck and hair,
And waved the broidered shawl that covered
Her taper bodice rounded fair ;

Or twirled the floating fringe that muffled
The pearly splendor of her arm,
And, dallying down her kirtle, ruffled
Its buoyant contour's silken charm,
To where, with coy reserve most wooing,
Glimmered the vision of her feet,
Like two coquetting pigeon's, cooing
Within and out their looped retreat.

She touched the earth with step as airy,
With port as gay and debonair,
As if the carol of a fairy
Had timed her motions to its air.
And, gliding on, she left me dreaming
How far more perfect in her face
The light of that young soul was beaming,
That lent her form such perfect grace.

The laborer paused, the merchant tarried,
By noisy street, by trade-vexed mart,
As if her very presence carried
A blessing to the jaded heart ;
And, though unprized the fleet enjoyment,
Each heart turned cheerier to its cares.
For each had entertained one moment
A wandering angel unawares.

She turned and vanished, but in going
Revealed a face where glad content
Through artless innocence was glowing,
And sweet reserve with archness blent ;
Not formed to dazzle by its splendor,
But through its winning softness beamed
A spirit constant, frank and tender,
That leaves no promise unredeemed.

She faded like a lovely vision,—
A joy undimmed by doubts or fears !
Falsehood, neglect, and dark suspicion
Waylay the garnered hopes of years,
Friendship and love and honor perish—
They perish, leaving venomed stings,
Alas, too late we learn to cherish
The chance delights the moment brings.

---

### RESIGNATION.

BY HENRY W. LONGFELLOW.

There is no flock, however watch'd and tended,
But one dead lamb is there!
There is no fireside, howsoe'er defended,
But has one vacant chair!

The air is full of farewells to the dying,
And mournings for the dead;
The heart of Rachael for her children crying
Will not be comforted!

Let us be patient! these severe afflictions
Not from the ground arise,
But oftener celestial benedictions
Assume this dark disguise.

We see but dimly through the mists and vapors;
Amid these earthly damps
What seem to us but dim, funeral tapers
May be Heaven's distant lamps.

There is no Death! what seems so is transition;
This life of Mortal breath
Is but a suburb of the life elysian,
Whose portal we call death.

She is not dead—the child of our affection—
But gone unto that school,
Where she no longer needs our poor protection,
And Christ himself doth rule.

In that great cloister's stillness and seclusion
By guardian angels led,
Safe from temptation, safe from sin's pollution,
She lives whom we call dead.

Day after day we think what she is doing
In those bright realms of air;
Year after year, her tender steps pursuing,
Behold her grown more fair.

Thus do we walk with her and keep unbroken
The bond which nature gives,
Thinking that our remembrance, though unspoken,
May reach her where she lives.

Not as a child shall we again behold her;
For when with raptures wild
In our embraces we again enfold her,
She will not be a child;

But a fair maiden, in her Father's mansion,
Clothed with celestial grace;
And beautiful with all the soul's expansion
Shall we behold her face.

And though at times, impetuous with emotion
And anguish long suppress'd,
The swelling heart heaves moaning like the ocean
That cannot be at rest;

We will be patient! and assuage the feeling
We cannot wholly stay;
By silence sanctifying, not concealing
The grief that must have way.

### THE CURIOUS WAYS OF PLANTS.

BY A. B. HARRIS.

Who can account for the ways of plants, or explain why a certain species will grow in one place, and will not in another exactly similar, so far as human intelligence can determine?

The American aloe is a hundred years in getting ready to flower; whereas, the gourd grows like Jack's bean-stalk. Some wild flowers disappear on the advance of civilization; while, on the other hand, the plantain—if the truth is told—goes wherever Europeans go; and, in this country, was unknown until after the English came—following so closely on their tracks that the Indians gave it the name of "white man's foot."

Some varieties, as above intimated, may be found in a particular locality, and nowhere else within half-a-dozen miles. There is, for example, in this neighborhood, in central New England, one spot where are a few shrubs of the mountain-laurel—"spoon-wood"—in a little patch by the roadside; and, although this would seem the natural country for it, it can be discovered in no other place anywhere about.

Then there is the fringed gentian, which has been seen beside a secluded road, some six miles away, but with that exception appears wholly unknown in the vicinity; yet the closed gentian is abundant. Another of the perversely disappointing flowers is the dog-tooth violet; not, however, more capricious than the yellow violet, and the noble liverwort, (*Hepatica triloba*), which in certain dry maple woods, in the one case, and in open knoll-covered pastures in the other, grow in great abundance; still one might search acres of similar woods and pastures for them, all to no purpose.

Another case, somewhat in point, is the holly—indigenous, or at least one variety, to moist woods along the eastern border of New England, but so partaking of the afore-named eccentricity, that he may count himself a happy man who can find it, and prove his success by great armfuls of it, wherewith to deck his house at Christmas. One gets glimpses of it while riding through some swampy tract on Cape Ann—the bright berries and evergreen leaves so suggestive of English good cheer, betraying it. There too, in summer, by searching diligently, one may find a species of magnolia—that being about its northern limit.

No common New England flower is so little to be depended upon as the trailing arbutus. It is difficult to determine what it wants. It abounds on gravelly knolls by the wayside, and thrives on the very edge of pasture bogs, and in the shade of woods; and yet, with all this versatility, there are many towns where it is never found, and where, though transplanted and tended with care, it cannot be made to live.

Quite opposite, in these respects, is the "cardinal-flower," whose home is by the water-side—the only place where it grows naturally, although the kind of water is not of imminent consequence, for it will do just as well in a dark nook under the up-heaved root of a willow, on the edge of a mill-pond, in the muddiest ooze, as in the cleanest sand along a river's bank—its chief requirement seeming to be that it shall not be crowded, one stalk always standing by itself, independent of its kind, and not in close neighborhood to other plants. It is so adaptive, that it will bear removal to a garden—taking kindly to its new conditions; and there it will come up, year after year, flaming out in live scarlet, in "one glorious blood-red," as if nothing had happened to it.

There are other facts, more singular, as to the ways of growth and hows of blooming. One can understand that a grape-vine may hold to its support by means of a tendril, while an ivy or a Virginia-creeper secures itself by thrusting its rootlets into a crevice of a wall or in the bark of a tree; but why should a honeysuckle and a bean-vine wind in opposite directions? the one goes to the left, and the other to the right; and both will swing on the wind, or sprawl over the ground, rather than turn the other way.

The ketmia opens at nine o'clock in the morning, and shuts at ten, as if it had a visual weakness; while a bed of portulaccas never expands unless the sun is out; and the hotter he shines, the wider they spread themselves; and the evening primrose waits until he has gone down, and then comes open with a snap, like a subdued kind of fire-cracker.

But most unaccountable of all, perhaps, is the night-blooming jasmine. You see a simple tree-like plant, with a plain style of leaf, at the base of which grows a spray of yellowish-green tubes, like lilac-buds—suggesting more than anything else a string of small candles. You look at them in the middle of the day, and they are "only that and nothing more;" and you might, if you did not know its ways, forget all about it; but when evening comes, forgetting is impossible. The room is full of fragrance, rich as orange-flowers, and almost as subtile as violets; and lo! your little candles are all lighted; and from somewhere about them comes that perfume which is so delicious and so mysterious as to its source. The next morning, they begin to contract; by noon, the five points are all close packed; and there is no scent to them or about them at all, till night comes on again; and so they continue, scentless through daylight, but of exquisite sweetness when darkness appears.

### GALILEO'S TELESCOPE.

In the year 1609, Galileo relates, he first heard from a friend in Flanders that an instrument had been invented by which distant objects were brought near and the powers of vision extended. He resolved at once to imitate and surpass it. By his singular mechanical dexterity, his knowledge of optics, and his highly polished glasses, he was soon able to produce a telescope before which the Dutch instrument sank into neglect and was forgotten. It was never more heard of; but a thrill of wonder passed over Italy and Europe when it was known that the Paduan professor had prepared an optic glass that enlarged the bounds of vision and endowed mankind with new powers. The charm of surpassing novelty covered the wonderful invention with an unprecedented renown. The great and the learned contended for the possession of the new instrument. Galileo carried his telescope to Venice, and from the tallest bell-towers senators and nobles saw through the magic glass great argosies sailing far out at sea, and the distant shores brought near and made visible. All the value of the new instrument broke at once upon their minds: it must change the principles of military strategy, and diminish the perils of navigation. Magistrates, senators, and citizens covered the fortunate inventor with applause. With discreet courtesy, Galileo presented his telescope to the Doge at a friendly audience, and the Venetians at once raised his salary to a thousand florins. Covered with honors and emoluments, he returned to Padua, little conscious of the surpassing discoveries that yet awaited him in the silent heavens, or of the pains and woes he was destined to bear in his later years from the heretical revelations of his too truthful and fatal telescope.—*Harper's Magazine for August.*

Harsh words are like the hail which beats
The herbage to the ground;
Kind words are like the gentle rain
Which scatters freshness round.

As polished steel receives a stain
From drops at random flung,
So does the child, when words profane
Drop from a parent's tongue.

The rust eats in, and oft we find
That naught which we can do,
To cleanse the metal or the mind,
The brightness will renew.

### THE LAW OF LONGEVITY.

At the meeting of the American Public Health Association, a carefully prepared paper was read by Dr. Nathan Allen, of Lowell, Mass., upon what he denominated the "Law of Longevity." In his essay some new and, if true, very important views were presented. This gentleman has devoted special attention for many years to physiology in its bearings upon the changes and increase of population, and is the author of several pamphlets upon this and kindred subjects, which have attracted much attention.

He maintains that nature has established a great law of increase, which applies not only to the human race, but prevails with modified conditions throughout the whole animal and vegetable kingdoms. This law is based in physiology upon the perfectionism of structure and harmony of function, or, in other words, that every organ in the system should be perfect in its structure, and that all should perform fully their respective functions in harmony with each other. Upon this same basis or foundation Dr. Allen places the law of longevity, and in the paper referred to adduced many striking facts and arguments in its favor, of which we can give only a brief synopsis. The existence of such a law is supported by all the well-known truths in Physiology and Pathology. Every change from a normal to an abnormal state, or in the prevention and cure of disease, affords evidence of such a law.

The pre-requisites to, or necessary conditions of longevity, Dr. Allen discussed under three heads: first, sound constitution; second, laws of inheritance; and third, obedience to the laws of hygiene. In order to secure good health and long life, a sound and well-balanced physical organization is found indispensable. But where is our guide or standard to test this soundness or balance? We have only approximations towards this standard, and a great diversity of opinions respecting them, because there is no universal type or perfect model upon which to base our judgments.

In some respects the human body, in its normal state, may be compared to a perfect machine made of many complicated parts. How different the working or running of such a machine from that of one imperfectly constructed and unequally balanced? The one seldom needs repairs; the other, frequently. It is so in reference to the body. Whenever a certain organ or class of organs are relatively too large or too small, or are exercised too much or not enough, causing a want of harmony in their action, there must be greater liability to disease. How often it happens that some slight derangement or trifling weakness operates as an entering wedge to the most serious and dangerous diseases? Hence the importance of a sound and well-balanced constitution, and the nearer the approximation can be made to it the better. This is indispensable, not only for good health, but for long life. But such a constitution can be secured only from long-lived ancestry. This accords with universal experience, as well as with all the principles of physiology. If we apply the well-known law, that "like begets like," to the healthiest families found, and, observe it through several generations, the result will be, that we obtain very sound and healthy constitutions.

Longevity is not dependent so much upon climate or food or employment as upon the physical organization itself. It is true these have a powerful influence upon health, but they are secondary agents. The general law exists in the body, and not outside. The laws of inheritance are a part of it; so are the principles of hygiene. It is not a mere theory or speculative hypothesis, but can be easily comprehended and applied. There is one place where it can be made of incalculable value, viz.: in the matter of life insurance. It furnishes the medical examiner a standard of organization with which the constitution of all persons applying for life insurance, can be compared, enabling him to judge very correctly what are the deviations from a normal standard; then, what are the liabilities to disease, and what are the probabilities of long life.

It points out the true sources and means of health and life, and that there is no chance or mystery in them. It shows that all the changes that occur in the human system are governed by law; that disease of whatever character, or wherever found, is a violation of law, and all treatment, whether provided by Nature or not, must be viewed as an agent to repair the injury. To describe all the various ways in which this law of longevity may be practically applied, would require, said the writer, a volume. The closing paragraph of this paper was as follows: It expounds correctly the great laws of inheritance which furnish the groundwork—the pre-requisite, for good health and long life. It teaches the absolute necessity in the outset of possessing a sound constitution—a well-balanced organization. It shows the relation of and importance which human agency holds in propagating a sound and healthy stock. It presents constantly before us for imitation that perfect standard and image in which man was created, together with an embodiment of those laws and conditions with which we must comply in order to secure the greatest amount of happiness and the longest duration of life. It teaches every individual more clearly what are the peculiarities and weaknesses of his own constitution, as well as what are his particular dangers or liabilities to disease. It is this exact, this definite and personal knowledge that may be turned to the greatest account in the preservation of health. If every individual could thus be made thoroughly acquainted with his own physiology, together with the laws of hygiene in his own case, we should soon see a most surprising diminution of sickness as well as of early mortality.

### PRACTICAL EQUALITY OF THE SEXES.

THE main factors of the relation between the sexes have hitherto been, and probably still are, natural affection—the man's need of a helpmate, the woman's need of a protector and provider, especially when she becomes a mother, and the common interest of parents in their children. One of these factors must be withdrawn, or greatly reduced in importance, to warrant us in concluding that a fundamental change in the relation is about to take place. Mr. Mill hardly notices any one of the four, and he treats the natural relation which arises from them as a purely artificial structure, like a paper constitution or an act of Parliament, which legislatures can modify or abolish at their pleasure.

It has no doubt been far from a satisfactory world to either sex; but unless we attach a fictitious value to public life and to the exercise of public professions, it will be very difficult to prove that it has been more unsatisfactory for one sex than the other. If the woman has had her sorrows at home, the man has had his wars and his rough struggles with Nature abroad, and with the sweat of his brow he has reclaimed the earth, and made it a habitation for his partner as well as for himself. If the woman has had her disabilities, she has also had her privileges. War has spared her; for, if in primitive times she was made a slave, this was better, in the days before sentiment at least, than being massacred. And her privileges have been connected with her disabilities. If she had made war by her vote, she could not have claimed special respect as a neutral, nor will she be able to claim special respect as a neutral if she makes war by her vote hereafter.—*Popular Science Monthly for August.*

### THE STRUGGLE FOR WEALTH.

No one can settle down in a European city or village for a month, and observe the laboring classes, without noticing a great difference between their aspirations, ambitions and habits, and those of corresponding classes in this country. He may see great poverty in a continental town, and men and women laboring severely and faring meanly, and a hopeless gap existing between classes; he may see the poor virtually the slaves of the rich; but he will witness a measure of contentment and a daily participation in humble pleasures to which his eyes have been strangers at home. There is a sad side to this pleasant picture. Much of this apparent contentment and enjoyment undoubtedly come from the hopelessness of the struggle for anything better. An impassable gulf exists between them and the educated and aristocratic classes—a gulf which they have recognized from their birth; and, having recognized this, they have recognized their own limitations, and adapted themselves to them. Seeing just what they can do and cannot do, they very rationally undertake to get out of life just what their condition renders attainable. There is no far-off, crowning good for them to aim at, so they try to get what they can on the way. They make much of fete-days, and social gatherings, and music, and do what they can to sweeten their daily toil, which they know must be continued while the power to labor lasts.

In America it is very different. A humble backwoodsman sits in the Presidential Chair, or did sit there but recently; a tailor takes the highest honors of the nation; a canal-driver becomes a powerful millionaire; a humble clerk grows into a merchant prince, absorbing the labor and supplying the wants of tens of thousands. In city, State and national politics, hundreds and thousands may be counted of those who, by enterprise, and self culture, and self-assertion, have raised themselves from the humblest positions to influence and place. There is no impassable gulf between the low and the high. Every man holds the ballot, and, therefore, every man is a person of political power and importance. The ways of business enterprise are many, and the rewards of success are munificent. Not a year, nor indeed a month passes by that does not illustrate the comparative ease with which poor men win wealth or acquire power.

The consequence is that all but the wholly brutal are after some great good that lies beyond their years of toil. The European expects always to be a tenant; the American intends before he dies to own the house he lives in. If city prices forbid this, he goes to the suburbs for his home. The European knows that life and labor are cheap, and that he cannot hope to win by them the wealth which will realize for him the dream of future ease; the American finds his labor dear, and its rewards comparatively bountiful, so that his dream of wealth is a rational one. He, therefore, denies himself, works early and late, and bends his energies, and directs those of his family into profitable channels, all for the great good that beckons him on from the far-off, golden future.

The typical American never lives in the present. If he indulges in a recreation, it is purely for health's sake, and at long intervals, or in great emergencies. He does not waste money on pleasure, and does not approve of those who do so. He lives in a constant fever of hope and expectation, or grows sour with hope deferred or blank disappointment. Out of it all grows the worship of wealth and that demoralization which results in unscrupulousness concerning the methods of its acquirement. So America presents the anomaly of a laboring class with unprecedented prosperity and privileges, and unexampled discontent and discomfort.

There is surely something better than this. There is something better than a life-long sacrifice of content and enjoyment for a possible wealth, which, however, may never be acquired, and which has not the power, when won, to yield its holder the boon which he expects it to purchase. To withhold from the frugal wife the gown she desires, to deny her the journey which would do so much to break up the monotony of her home-life, to rear children in mean ways, to shut away from the family life a thousand social pleasures, to relinquish all amusements that have a cost attached to them, for wealth which may or may not come when the family life is broken up forever—surely this is neither sound enterprise nor wise economy. We would not have the American laborer, farmer and mechanic become improvident, but we would very much like to see them happier than they are, by resort to the daily social enjoyments which are always ready to their hand. Nature is strong in the young, and they will have society and play of some sort. It should remain strong in the old, and does remain strong in them, until it is expelled by the absorbing and subordinating passion for gain. Something of the Old World fondness for play, and daily or weekly indulgence in it, should become habitual among our workers. Toil would be sweeter if there were a reward at the end of it; work would be gentler when used as a means for securing a pleasure which stands closer than an old age of ease; character would be softer and richer and more childlike, when acquired among genial, everyday delights The all-subordinating strife for wealth, carried on with fearful struggles and constant self-denials, makes us petty, irritable and hard. When the whole American people have learned that a dollar's worth of pure pleasure is worth more than a dollar's worth of anything else under the sun; that working is not living, but only the means by which we win a living; that money is good for nothing except for what it brings of comfort and culture; and that we live not in the future, but the present, they will be a happy people—happier and better than they have been. "The morrow shall take thought for the things of itself," may not be an accepted maxim in political economy, but it was uttered by the wisest being that ever lived in the world, whose mission it was to make men both good and happy —*Dr J. G. Holland; Scribner's for August.*

---

### PINCHBECK JEWELRY.

### MODERN ALCHEMY AND ITS ACHIEVEMENTS.

People who make any pretensions to being fashionable will wear glittering ornaments somehow, and prefer the false to none at all. The art of imitating the genuine has become so perfected that, apart from certain tests which in ordinary circumstances it is convenient or impossible to apply, not one in ten thousand can detect the difference. In dash and splendor the imitated are often scarcely inferior to the originals, whence by the chemist's magic they are copied.

In the manufacture of

ARTIFICIAL DIAMONDS, the object of the splendid illusion is to produce a perfectly colorless substance, thoroughly lucid, and capable of reflecting all lights. First, it is necessary to dissolve charcoal. Then follows processes requiring crystallization—a mingling of pure water, a little carbonate of sulphur, and certain proportions of liquified phosphorus. Still, all this may not yield a thoroughly deceptive diamond. Another composition is made from silver sand, very pure potash, minium, calcined borax, and a form of arsenic, varied occasionally by a mixture of *strass*—a mixture for which an equivalent is *paste*, and which represents transparent pebbles burnt to powder, white lead, and other similar materials. Sometimes rock-crystal is used, with borax acid from Italy, and nitrate of potash.

Of these materials are composed the false diamonds which figure so alluringly in the shop windows.

THE SAPPHIRE

is a product of the East, though found of inferior quality in Bohemia, Saxony, and France, among the rocks of the secondary period. There are white sapphires, occasionally mistaken for diamonds : crimson or carmine, resplendent beyond description; vermilion and topaz-tinted. Indeed, we may assign rank to the emerald as daughter of the sapphire. But the imitation of each and all of them is simple enough, if you but know how. Take, as the cookery-books say, one ounce of paste, mix with two grains of precipitated oxide of cobalt, and there you have the colored and glowing necklet, which none except a jeweler can detect. Coming to the splendid gem,

THE RUBY, whether of Brazil, Barbary, or Bohemia, with its cherry of purple red, varied by opalescent or milky aspects, there are various methods of rivaling it—with litharge and calcined shells ; with paste, antimony, glass, and purple of Cassius ; with white sand washed in hydrochloric acid, minium, calcined potash, calcined borax, and oxide of silver stirred in a crucible.

THE TOPAZ, or something very like it, can be obtained from a little white lead with some shells of a rich tint pulverized and calcined ; or from a mixture of antimony, glass, and ordinary jeweler's paste with purple of Cassius ; or, again, by a composition of white lead, minium, burnt potash, burnt borax, and oxide of silver.

EMERALD, AMETHYST, ONYX, & CORAL.

Each in turn is imitated by the skill of the chemist in combining materials of comparatively easy procurement. There is a notorious manufacture of onyx nearly all over Europe, from German pebbles, treated with acids ; and the false can scarcely be distinguished from the true, except by their weight and price. We should recommend very great caution in purchasing what purports to be onyx. In no kind of precious stones is more deception practiced.

FALSE PEARLS

were invented in Paris towards the close of Henry IV.'s reign by an ingenious fellow named Jaquin. Thence the manufacture spread into Italy, where it was extensively practiced, though the French specimens retained their superiority.

THE ROSE PEARLS OF TURKEY

are formed by pounding fresh and young flowers in a mortar until they become a paste, spreading this on cloth, and laying to partially dry in the sun. When nearly dry, they are pounded again in rose water, and dried again, and so on until the paste is exceedingly fine, when it is rounded into shape, polished with rose-water, for the sake of lustre and scent, and thus become the pretty impostures celebrated as the rose-pearl.

---

### RELATIONS OF ANIMALS AND PLANTS.

The animal, therefore, takes from the air oxygen, and turns it into carbonic acid ; the plant takes that carbonic acid, and turns it back into oxygen, which has thus discharged the great office of carrying carbon from the bodies of animals, and transferring it to the systems of plants. In what an interesting relation do the two kingdoms, the animal and the vegetable, thus stand to one another, not alone as respects the air in maintaining its constitution uniform by a mutual antagonization, but also as respects their own structures ! The elements of which plants are formed have all been derived from the pre-existing parts of animals ; and the elements of which animals consist, from the pre-existing parts of plants. To the classical scholar, what a beautiful commentary on the fictitious stories of antiquity are these modern discoveries ! He calls to mind the metamorphoses that

Ovid describes; the bore, perhaps, of his school-boy life, the elegant amusement of his later years. He remembers how Daphne was turned into a laurel, and Adonis into a flower; the musical stanzas are no longer an empty sound, they are descriptive histories. The thing he has read of is actually so. These transformations, instead of being imaginary exceptions, are the common lot of life in this world. There grows not now a leaf that is not formed from the parts of animals that are dead; there lives not a solitary animal being which has not derived its constituent elements from plants.—*From "Priestley's Discovery of Oxygen," in Popular Science Monthly for August.*

---

### THE TRUE METHOD OF CONSTRUCTING BOOTS, SHOES AND LASTS.

*A Paper read by Joel McComber before the Polytechnic Branch of the American Institute, New York, May* 14, 1874.

Mr. President and Gentlemen,

While there is no portion of the body more interesting and instructive, there is no part more neglected or less understood, than the feet; ample provision is made for every other member of the body, both with regard to comfort and appearance. Who wears a coat which draws, pinches or constrains any part of the form? Who wears a hat which causes pain or even discomfort to the head? Who wears a shirt for a single day which does not fit and feel easy and comfortable? But it is a well-known and indisputable fact, that a large portion of our population wear boots and shoes which are a source of constant annoyance and even at times of excruciating pain. Who among us having feet in the least disposed to be tender does not dread breaking in a pair of boots? Now, wherein lies the reason, that while the head, trunk, hands and limbs are well and comfortably protected, the feet are slighted, if not absolutely neglected? Why is it that a gentleman who can supply himself with every luxury, comfort and delight this world affords, cannot provide himself with the comfort of a well-fitting shoe? Now, I answer:

First.—The reason is not because men are not willing to pay sufficient for this article of clothing. It cannot be denied that fashionable shoemakers charge and receive perfectly adequate, and in many cases exorbitant, prices for making boots and shoes to order.

Second.—It is not because shoemakers do not really and honestly try to satisfy their customers.

Third.—It is not because a shoe properly made is offensive to our ideas of beauty and symmetry.

The great reason why lasts, boots and shoes do not fit feet is because there is among last and shoemakers no method system or principle upon which lasts or shoes are made. I am stating a simple, incontrovertible fact when I say that lasts and shoes are made wholly by guess work. Suppose a ship leaves this port to-day for Brazil; the captain says: "Now I know Brazil is south of here, and the temperature averages at this time of the year 90° Fahrenheit; I will therefore keep sailing southerly until my thermometer indicates 90°, and then I shall reach Rio Janario." Would he arrive at the desired port? Yet he would have more method in his madness, and would be more likely to attain to his desire than the last maker of the present day, who starts out in the endeavor to make a last to fit the foot, which he gravely and sagely measures.

Why, gentlemen, if we showed no more science in shoeing horses than we do in shoeing men and women, a horse traveling a mile in three minutes would be a nine days' wonder, and Robert Bonner's occupation would be gone. Now, I have the pleasure of exhibiting to you to-day lasts and shoes which are made to fit feet, and which do fit them.

And I make this assertion, viz. : That shoes can be made to fit any foot, great or small, from the flat foot to the aristocratic pedal extremity, which will allow a stream of water to flow under the instep without wetting the skin ; and when these shoes are put on they require no breaking in whatever, any more than a pair of stockings does. All breaking-in has been already performed, not by some person's foot, but by the last itself. And whether the foot has a hide like that of an ox, or a skin as soft and tender as an infant's, the shoe will be as easy the first week of wearing, or the first day, as an ordinary shoe is during the last week, or last day it is worn, and at the same time does not injure the foot the slightest; while an ordinary shoe, however old and apparently easy, is continually injuring and mis-shaping the foot. And, let it be fully understood, that boots and shoes made under this system are of fair proportions and symmetrical shape, and are not ugly nor offensive to our ideas of beauty. I do not overlook the fact that attempts have been made, and patents granted, for the purpose of remedying this evil ; some have been in the form of immensely wide soles, awkward in shape, tiresome and injurious to the feet. Anatomical lasts, so called, have been constructed, planned upon some idea of human anatomy unknown to the Creator, and to be found only in the visionary brains of the inventor. The truth is, that feet are expected to accommodate themselves to the shoes instead of the shoes being made to fit the feet. The object of my invention, for which I have secured letters patent, is an entirely new method or system of making lasts, boots and shoes ; is to produce a shoe which will conform to the anatomical structure of the human foot, and which will fit it snugly, but comfortably in whatever position the foot is placed, and especially when the pressure of the weight of the body is upon it. My last has an outside ball and shank, projecting laterally more than is usual in ordinary lasts.

The shank is lowered also in order to conform more closely to the shape of the foot.

The instep is located upon a line drawn from the centre of the heel to the inside of the great toe, as it is in the human foot, and overhangs the sole. The inner ball of the last overhangs the sole. The inside shank or hollow of the last is likewise made to conform to the shape of the foot. The tendency of the foot when walking or standing is to spread outwardly, so that it will be natural that shoes made upon the usual lack of method bulge and spread on the outside over the edge of the sole, so that a portion of the weight of the body rests in the bulged upper, instead of upon the sole. The sole is not under the whole foot.

Now, in boots and shoes made upon my last, the sole is, so to speak, pushed along sideways under the foot, each sole being moved outwardly to the place where the foot will be when the weight of the body spreads it. As shoes are made now, with the instep ball and shank totally misplaced, human feet are distorted until one would suppose that the shoemakers and lastmakers had combined into an organization intended to improve on the original foot as designed by the Creator. Without any desire to exaggerate facts, I should say, after thirty-two years of experience and observation as a practical lastmaker and shoemaker, that, in my opinion, a perfectly formed foot upon a person arrived at maturity is a rare exception. And, when we reflect how these deformities are transmitted from generation to generation, it does seem a sin and shame that we cannot better preserve our bodies, so beautifully and wonderfully made, in their original beauty. What is there prettier than an infant's foot ? What is there uglier than that same foot distorted and covered with blemishes and excresences after thirty or forty years of handling by ignorant shoemakers ? Where is the fashionable woman now "who walks a Goddess and who moves a Queen ?" Go among the uncivilized nations of the earth, and, if the missionaries have not been there too long, you will find more perfect feet than in our cultivated country. It may not be generally known that the condition of the feet has a great and marked influence upon the health. There is hardly a portion of the body which may not be affected by the state of health or disease in the feet. Many diseases, some incurable, proceed directly from the feet, and all physicians agree in recommending the utmost care of the lower extremities. How many lay the foundation of sickness and deformity in the feet of their children who would be indignant if anyone should ever hint to them that they were not entirely devoted to the interest of their little ones? It is hardly possible for me, unaided by diagrams, to describe in detail the nature and operations of my method.

Suffice it to say, that it is novel, revolutionary, and entirely successful, and I shall be happy to answer any questions which may be propounded with regard to it. I might dwell upon the moral aspects of the case, and assert what, although it might excite a smile, would be none the less true, that as much profanity, ill-temper, peevishness, and kindred vices, with all their "consequential damages," have been produced by ill-fitting boots and shoes as from any other known cause.

But I will merely close this brief statement by recommending all who desire comfort in this life, and wish it said of them "how beautiful upon the mountains are their feet, etc.," to aid in promoting this new dispensation, and thus be true philanthropists as well as lovers of nature.

---

[The greatest of family blessings is a shoe made from McComber's "last," invariably giving the most natural, easy and perfect fit in the world.—Dr. W. W. Hall, Editor Journal of Health.]

---

### EDITORIAL NOTICES.

McComber's Patent Last and Pattern Boots and Shoes.—We have found from personal experience that a Boot or Shoe made on McComber's Patent Last fits with such perfect ease and comfort at the first trying on, that if it were generally known, the demand would soon be almost universal, and deservedly so, for it is conducted on principles strictly scientific and physiological, hence, every foot may be exactly and easily fitted at the very first trial.

Address Joel McComber, No. 21½ Spruce Street, New York. Descriptive Pamphlet free.

---

The *New York Tribune* has lately published in its "extra," No. 20, two essays of great practical value to medical students, and all seekers after Nature's laws of health. They are Ex-Surgeon General Hammond's lecture on "Alcohol as Food, and Dr. Brown Sequard's on the "Brain and Nerves," each of which contains the latest and freshest fruit of scientific research on these subjects. The price of this extra is twenty cents, and may be obtained by sending that sum to the office of *The Tribune*, which we regard as truly "the leading American newspaper."

---

"The Mineral Springs of the United States and Canada," by George E. Walton, M.D., published by Appleton & Co., is a work we can cordially recommend to our readers as conveying much valuable information in an attractive form. It treats of the history, action, chemical constituents and curative properties of the mineral springs and baths, and advises as to their applicability to various diseases. Suggestions are also given as to the choice of health resorts. Some chapters are devoted to sea-bathing and the European Spas. It is now generally admitted, that most diseases may be benefited by the proper use of mineral waters. This book will prove a useful guide to those in pursuit of health.

